# Editorial: Refining precision medicine through AI and multi-omics integration

**DOI:** 10.3389/fgene.2026.1851372

**Published:** 2026-06-09

**Authors:** Viola Bianca Serio, Pu-Feng Du, Elisa Frullanti, Maria Palmieri

**Affiliations:** 1 Cancer Genomics and Systems Biology Lab, Department of Medical Biotechnologies, University of Siena, Siena, Italy; 2 Department of Medical Biotechnologies, Med Biotech Hub and Competence Centre, University of Siena, Siena, Italy; 3 School of Computer Science and Technology, Tianjin University, Tianjin, China

**Keywords:** AI, machine learning, omics, precision medicine, systems biology

## Introduction

The paradigm of modern healthcare is undergoing a profound transformation, shifting from a “onesize-fits-all” approach toward a more tailored, individual-centric model. The goal of this evolution lies in the synergistic convergence of high-dimensional multi-omics data and advanced artificial intelligence (AI) methodologies (Johnson et al., 2021). This Research Topic, “Refining Precision Medicine through AI and Multi-omics Integration,” brings together a Research Topic of innovative studies exploring how machine learning can decode the integration of genomics, transcriptomics, proteomics, and metabolomics to unlock new clinical insights. While the explosion of biological data has provided an unprecedented map of human diseases, the extreme complexity of these interconnected datasets makes it difficult to identify patterns that elude traditional statistical analysis.

In this context, the challenge lies not only in the sheer volume of “big data” but also in its intrinsic heterogeneity and the need for harmonization. AI methodologies, particularly deep learning and crossomic integration algorithms, emerge as indispensable tools for overcoming experimental noise and biases, enabling the mapping of non-linear relationships between genotype and phenotype (Eras et al., 2019). By creating integrated biological networks, researchers can identify hidden molecular drivers regulating disease progression, paving the way for a holistic understanding of human biology. This approach not only refines our predictive capabilities but also redefines the boundaries of medical taxonomy, allowing for disease classification based on molecular mechanisms rather than merely clinical symptoms (Hulsen et al., 2019).

The scientific contributions featured in this editorial highlight the pivotal role of AI in bridging the gap between raw molecular data and clinical significance. From deep learning autoencoders used for patient stratification to graph neural networks that model intricate biological interactions, the research presented here demonstrates significant leaps in diagnostic accuracy and therapeutic targeting. Key themes include the discovery of novel biomarkers for chronic diseases, the optimization of drug repurposing pipelines, and the development of predictive models that account for individual genetic variability. Furthermore, this Research Topic addresses critical challenges such as data standardization, model interpretability, and the ethical imperatives of privacy-preserving federated learning. By fostering multidisciplinary collaboration among computational scientists and clinicians, these articles collectively provide a roadmap for the next-generation of precision medicine, where AI-driven insights translate directly into improved patient outcomes and more resilient healthcare systems.

## The convergence of multi-omics and artificial intelligence

This Research Topic features 8 research articles, 1 review, 1 study protocol, and 1 research article correction regarding multi-omics approaches and AI integration.

Yaohui Wang et al. combined bioinformatics analysis with experimental validation to map the mitochondrial genetic landscape in pre-eclampsia, identifying key molecular drivers of the condition. Their findings highlight critical biomarkers and functional pathways linking mitochondrial dysfunction to pathogenesis, offering new insights for early diagnosis and targeted therapeutic interventions.

Han et al. utilized a multi-algorithm machine learning framework, specifically LASSO, SVM-RFE, and Random Forest and molecular dynamics to delineate the mitophagy-related landscape in ulcerative colitis. By identifying key biomarkers (CD55, CPT1A, and SLC16A1) and potential therapeutic agents like galunisertib, the research bridges the gap between mitochondrial dysfunction, immunometabolic reprogramming, and precision pharmacology.

In the race to develop more accurate computational models for drug discovery, Xing and Zhao studied the DDA-Bench as a timely solution, centralizing vital resources and baseline performances that were previously scattered across the literature. By accounting for variables like dataset density, this new database not only saves precious research hours but also sets a higher standard for how we evaluate and report drug-disease association models.

An interesting study was published by Zhou et al. about the integrative multi-omics approach to investigate the link between mitochondrial oxidative phosphorylation (OXPHOS) dysfunction and schizophrenia. Through transcriptomic analysis, WGCNA, and machine learning, the researchers identified and validated three key hub genes, MALAT1, PPIL3, and ITM2A, as potential regulators of ATP-generating pathways. These findings, corroborated by *in vivo* mouse models and singlenucleus RNA sequencing, highlight cell-specific metabolic deficits in excitatory neurons. Ultimately, the study identifies these genes as crucial biomarkers and therapeutic targets to address the bioenergetic alterations associated with the disease.

Through an integrative bioinformatics and machine learning approach, Least Absolute Shrinkage and Selection Operator (LASSO) and Random Forest (RF) Yi et al. identified ABO as a key hub co-diagnostic gene linking Crohn’s disease (CD) and osteoporosis (OP). Analysis of GEO datasets and *in vitro* validation revealed that *ABO* is significantly downregulated in both conditions, showing strong diagnostic potential and correlations with immune cell infiltration (e.g., CD8 T cells). These findings suggest that *ABO* plays a critical role in the shared molecular mechanisms of inflammation and bone resorption. Ultimately, this research provides a novel genetic biomarker for the co-management and clinical prediction of these overlapping pathologies. This study was corrected with the following publication: “Correction: Identification of shared diagnostic genes between osteoporosis and Crohn’s disease through integrated transcriptomic analysis and machine learning.

Li et al. introduced MKDR (Multi-Omics Modality Completion and Knowledge Distillation for Drug Response Prediction), a deep learning framework designed to handle missing multi-omics data in predicting drug responses for cervical cancer. By integrating variational autoencoders for modality completion and knowledge distillation, the model achieves state-of-the-art accuracy, significantly outperforming traditional methods like XGBoost. Interpretability analysis confirms a balanced integration of gene expression, copy number variation, and mutation data, maintaining high performance even with 40% data missingness. Ultimately, MKDR offers a robust and clinically viable tool for enhancing personalized treatment strategies in oncology.

Ren et al. developed a machine learning framework to predict cognitive impairment in aging populations by integrating urinary metal concentrations with demographic data. In this study, six machine learning algorithms, including Gaussian Naive Bayes (GNB), Random Forest (RF), K Nearest Neighbors (KNN), Support Vector Machine (SVM), Lasso and eXtreme Gradient Boosting (XGBoost) were employed to estimate the risk of cognitive dysfunction. By identifying specific trace element profiles associated with neurodegeneration, the research provides a non-invasive, cost-effective diagnostic tool for early screening and personalized risk assessment in geriatric care.

Finally, the machine learning (LASSO regression and random forest) was used by Gong and An to perform an integrated transcriptomic analysis to map the molecular evolution of COVID-19 from acute infection through the recovery phase. By employing LASSO regression and random forest and functional enrichment, stagespecific gene signatures and immune features were identified. The study defines robust diagnostic biomarkers that distinguish disease severity and track immunological restoration, providing a molecular roadmap for monitoring long-term post-viral outcomes.

Zheng et al. provided for this Research Topic a review that explores the integration of endocrine-metabolic profiling and artificial intelligence to refine the diagnostic and therapeutic landscape of pituitary neuroendocrine tumors (PitNETs). By utilizing AI to decode complex hormonal signatures and tumor heterogeneity, the research addresses current limitations, such as data fragmentation and model interpretability, providing a roadmap for multimodal data fusion and AI-driven precision management in neuroendocrine oncology.

The Research Topic is also completed with the publication of a study protocol from Zhang et al. regarding a multicenter prospective cohort study that addresses clinical discrepancies in Neonatal Acute Respiratory Distress Syndrome (NARDS) by integrating deep phenotyping with multi-omics profiling. By leveraging machine learning, including penalized logistic regression (Elastic Net), decision trees, random forests, gradient boosting machines, support vector machines, and artificial neural networks to analyze granular clinical data and longitudinal biospecimens from over 1,000 neonates, the research aims to develop highaccuracy predictive models for disease severity and long-term neurodevelopmental outcomes, paving the way for regional and individual-specific precision interventions in neonatal intensive care.

## Conclusion

In conclusion, the diverse contributions to this Research Topic underscore the essential role of integrative multi-omics and artificial intelligence in modern medicine. From discovering novel biomarkers in neurodegenerative and inflammatory diseases to developing resilient deep learning frameworks like MKDR, these studies bridge the gap between complex biological data and clinical utility. By addressing critical challenges such as data missingness and the need for standardized benchmarking, this Research Topic provides a comprehensive roadmap for precision medicine. Collectively, these findings not only refine our understanding of disease pathogenesis but also pave the way for more accurate diagnostics and personalized therapeutic interventions across diverse medical fields. As we move forward, the continued dialogue between bioinformaticians, clinicians, and ethicists will be paramount to ensuring that AIdriven insights are implemented responsibly and accurately. Ultimately, the integration of these advanced computational tools marks a critical step toward a future where healthcare is not only reactive but preemptive, ensuring that the promise of personalized medicine translates into tangible benefits for patients worldwide.
